# Stakeholder views on secondary findings in whole-genome and whole-exome sequencing: a systematic review of quantitative and qualitative studies

**DOI:** 10.1038/gim.2016.109

**Published:** 2016-09-01

**Authors:** Michael P. Mackley, Benjamin Fletcher, Michael Parker, Hugh Watkins, Elizabeth Ormondroyd

**Affiliations:** 1 Division of Cardiovascular Medicine, Radcliffe Department of Medicine, University of Oxford, Oxford, UK; 2 Nuffield Department of Primary Care Health Sciences, University of Oxford, Oxford, UK; 3 Ethox Centre, Nuffield Department of Population Health, University of Oxford, Oxford, UK

**Keywords:** ethical, legal, and social issues, genomics, genetic counseling, secondary findings, stakeholder perspectives

## Abstract

**Purpose:**

As whole-exome sequencing (WES) and whole-genome sequencing (WGS) move into routine clinical practice, it is timely to review data that might inform the debate regarding secondary findings (SF) and the development of policies that maximize participant benefit.

**Methods:**

We systematically searched for qualitative and quantitative studies that explored stakeholder views on SF in WES/WGS. Framework analysis was undertaken to identify major themes.

**Results:**

Forty-four articles reporting the views of 11,566 stakeholders were included. Stakeholders were broadly supportive of returning “actionable” findings, but definitions of actionability varied. Stakeholder views on SF disclosure exist along a spectrum: potential WES/WGS recipients' views were largely influenced by a sense of rights, whereas views of genomics professionals were informed by a sense of professional responsibility. Experience with genetic illness and testing resulted in greater caution about SF, suggesting that truly informed decisions require an understanding of the implications and limitations of WES/WGS and possible findings.

**Conclusion:**

This review suggests that bidirectional interaction during consent might best facilitate informed decision making about SF and that dynamic forms of consent, allowing for changing preferences, should be considered. Research exploring views from wider perspectives and from recipients who have received SF is critical if evidence-based policies are to be achieved.

*Genet Med* **19** 3, 283–293.

As genome sequencing moves from the realm of research and difficult-to-solve clinical cases into routine clinical care, so do the wider implications of this technology. In patients or families with a suspected genetic disorder for which single-gene or panel testing fails to provide a genetic explanation, genome sequencing is becoming widely available. Whole-genome sequencing (WGS) and whole-exome sequencing (WES)—wherein the genome or the protein-coding parts of the genome are sequenced in its entirety—might completely replace panel tests in the foreseeable future.

Although WES and WGS provide a valuable opportunity to learn about genomic contributions to disease (“primary” findings), they also have the potential to reveal genetic information that may not pertain to the patient's presenting condition, including variants associated with other health conditions, which may or may not be medically actionable and are unexpected.^1^ A variety of terms have been applied to such findings (“secondary,” “incidental,” “additional”);^2,3,4^ the term secondary findings (SF) has gained traction and is used here inclusively to cover findings that are not pertinent to the presenting condition, whether detected incidentally or actively sought.

The issue of SF management in WES/WGS has sparked much debate. SF are not new to medicine, but they are becoming a more frequent issue in genetics owing to more extensive use of WES/WGS in the diagnosis of rare disease and cancer. With each human genome expected to contain approximately 100 genuine loss-of-function variants,^5^ SF in WES/WGS are inevitable. Full disclosure of SF is economically and logistically impractical, but it has been argued that complete nondisclosure is unethical due to the potential benefits of medical intervention for some SF.^6^ Current practice in research and clinical settings varies between the two extremes. In 2013, the American College of Medical Genetics and Genomics (ACMG) put forward recommendations concerning SF in clinical WES/WGS: active screening of a defined list of genes, in which mutations could imply risk of potentially life-threatening disease, where intervention is available, and in which a long asymptomatic phase may conceal disease expression.^7^ The ACMG report suggested that in all individuals undergoing clinical WES/WGS, there is a moral obligation to report variants of known (or in some cases expected) pathogenicity and to interpret them in the context of the patient's personal and family history. Although there is support for the ACMG recommendations, opponents (and alternative guidelines from Europe and Canada) argue for greater caution in the analysis and return of such findings.^8,9,10,11,12^

As WES and WGS begin to be implemented in routine clinical practice, such as through the 100,000 Genomes Project currently under way in the UK National Health Service, increasing numbers of patients and their healthy relatives are being recruited. It is therefore timely to collect and review data that might inform this debate so that WES and WGS initiatives maximize clinical utility and contribute to ethical health-care delivery.

Commentators have argued that the development of policy around SF needs to consider the views and attitudes of relevant stakeholders and to obtain insights into the factors that have contributed to their formulation.^13^ Since the publication of a systematic review on secondary findings in genomics 4 years ago,^14^ which urgently called for research in this area, many empirical studies have been conducted. Due to the contemporaneous collection of these empirical data, SF management policies in WES/WGS have inevitably been unable to make full use of them. To date, we are aware of no systematic review of these studies; therefore, we have collated and synthesized them to evaluate the extent to which they might inform policy on secondary findings and to identify areas for further research.

## Materials and Methods

### Literature search strategy

**Figure 1:**
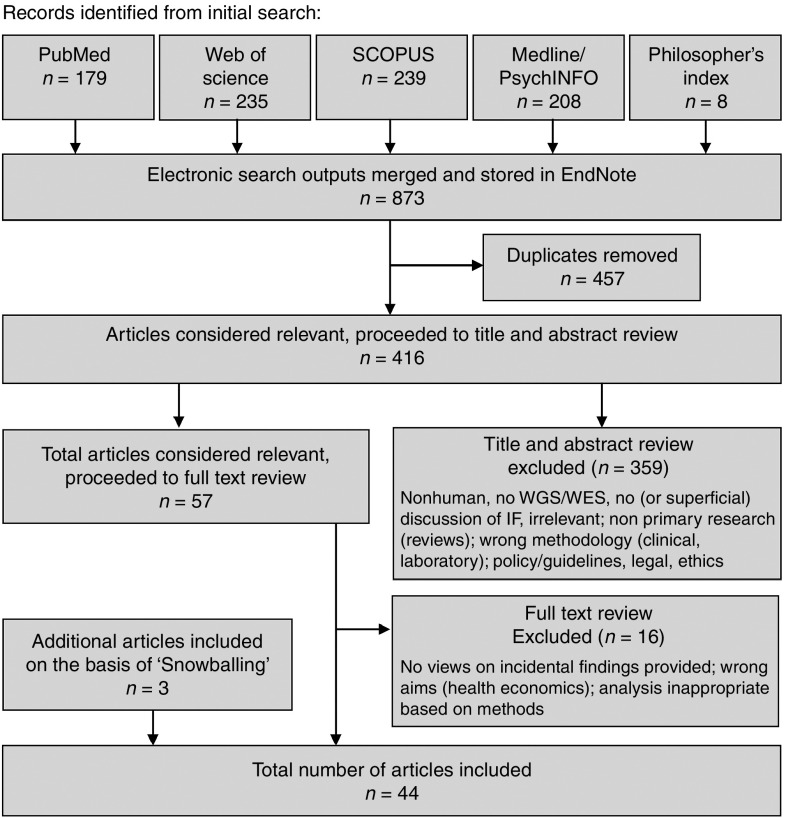
**Study selection process.**

We conducted a systematic search for research studies that examined views and preferences toward SF in WES/WGS. The protocol for this review was prospectively registered in an online database.^15^ Searches were conducted across six databases and included all literature in the databases from their date of inception to April 2015, with electronic updates included through 1 May 2016 (**Figure 1**). Search terms for secondary findings (including incidental and additional) were combined with terms for clinical sequencing (genetic, genomic, genome, and exome sequencing) (**Supplementary Table S1** online).

### Study selection criteria

The review included primary research articles based on qualitative (semi-structured interview-based and focus group-based) and quantitative (survey-based and questionnaire-based) methods. Studies were included if they explored stakeholder views and experiences regarding SF in WES/WGS in a health-care context in both clinical and research settings. Articles were excluded if they were not published in English or if the citation lacked an abstract. Articles were also excluded if they did not discuss WES/WGS (e.g., discussion limited to chromosomal microarray) or if they did not include (or only included superficial) discussion of secondary findings. Articles that explored views on return of results in a non–health care context, such as direct-to-consumer genetic testing, were also excluded. Secondary findings specifically for minors are not discussed because we acknowledge that this raises additional issues and could warrant their own review.

Two authors (M.P.M., E.O.) independently screened the titles and abstracts of all identified articles against the eligibility criteria using Covidence systematic review management software (http://covidence.org). In cases of disagreement, inclusion was resolved at a meeting between the two reviewers. Both reviewers then screened full-text copies of the selected articles to determine final inclusion. Initial screening was followed by reference scanning and snowballing, where the references of included works were searched manually to find additional articles relevant to the review.

### Data extraction and analysis

For both qualitative and quantitative studies, a general data extraction form was used that included bibliographic information, study characteristics, participant characteristics, and main findings. Two authors (M.P.M., B.F.) independently completed the data extraction for each study and one (M.P.M.) checked for accuracy and completeness.

A framework analysis approach was used^16^ whereby data integration occurred at the point of extraction. The approach was well-suited for this review because it can be applied to mixed methods studies and is particularly useful for multidisciplinary health research teams.^17^ Although traditionally applied to primary data, we used it as a review methodology. This was feasible because the studies investigate the same topics in a manner that can be categorized together.^17^

Initially, M.P.M. and E.O. reviewed six articles (three qualitative and three quantitative studies). The results sections of the qualitative studies were coded in a nonrestrictive manner and combined with analysis of questions asked across quantitative studies in order to produce a working analytical framework. This framework was applied to all of the other studies in which the results sections were coded (by M.P.M.) using NVivo 10 (QSR International) and manually reviewed (by E.O.). Throughout this process, the authors continued to revise the framework to reflect the data most accurately. Articles included from electronic updates were each coded (by M.P.M. and E.O.) and integrated into the preexisting framework. Data from the final framework were compared and evaluated to derive a higher-order synthesis that goes beyond the content of the included articles.

### Critical appraisal

Each of the studies was assessed using an established quality appraisal system, with a particular emphasis on assessing the comprehensiveness of reporting. In the case of the qualitative studies we used the COREQ checklist tool,^18^ which was adapted using the Critical Appraisal Skills Programme checklist (**Supplementary Table S2** online).^19,20^ In the case of the quantitative studies, we used a checklist tool adapted from Boynton and Greenhalgh^21^ and Greenhalgh et al. (**Supplementary Table S3** online).^22^ In both cases, two authors (M.P.M. and B.F.) independently scored articles according to the appropriate checklist after piloting three by consensus. Interassessor reliability was measured by calculating κ statistics. No quality cut-off was used, but a set of initial screening questions was used to ensure appropriateness of methodology and analysis and relevance to synthesis topic .

## Results

### Systematic review

A total of 44 published research studies met the criteria for inclusion (**Supplementary Table S7** online). Of the included articles, 15 used quantitative methods, 25 used qualitative methods, and 4 used mixed methods (**Table 1**). The majority of the studies (72.7%) were from the United States, with the others representing Canadian (13.6%), European (9.1%), and multinational (4.5%) groups of stakeholders.

### Study participants

Accounting for studies reporting on the same participants, the viewpoints of 11,566 unique stakeholders are represented (**Table 1**). Stakeholders include patients with genetic disorders or cancer diagnosis, relatives, WES/WGS research participants and members of the public (collectively termed “WES/WGS recipients”); health-care professionals (HCPs) in genetics or other specialties, both patient-facing and health-care scientists, primary care physicians, genetics researchers, and institutional review board chairs (“WES/WGS providers”). Both of these groups include actual and potential recipients or providers of WES/WGS. Participants have a wide range of exposure to genetic disease and genetic testing, from potentially no direct exposure (members of the public) to those who are actively providing or receiving WES/WGS; 34.1% of studies include participants who have “direct WES/WGS experience” (providing or receiving) together with participants who do not (**Table 1**). No studies in the review focus on stakeholder experiences with handling or receipt of genomic SF.

### Critical appraisal

Across the studies, quality and comprehensiveness of reporting were highly variable (**Supplementary Tables S4 and S5** online): scores for quantitative papers ranged from 8 to 23 (out of 26), whereas scores for qualitative papers ranged from 11 to 19 (out of 32). Although reporting of data collection was weak across the studies, with few qualitative studies reporting the theory underpinning analysis, findings/results were generally well reported. Qualitative studies elicited novel concepts and acted as particularly rich sources of data for the synthesis. Between reviewers, there was substantial agreement on the scores for quantitative papers (κ score = 0.614, 95% CI: 0.538, 0.690) and qualitative papers (κ score = 0.749, 95% CI: 0.697, 0.781).

### Thematic areas

From the framework analysis we identified 10 major thematic areas (**Table 2**). Illustrative quotations from the primary studies can be found in the **Supplementary Material and Methods** (**Table S6** online).

*Preferences for secondary findings.* An overwhelming majority of stakeholders believe that some form of SF should be returned if identified.^23,24,25,26,27,28,29,30,31,32,33,34,35,36,37,38,39,40,41,42,43,44^ When measured quantitatively, studies report a high desire (95–100%) to receive or return clinically actionable SF^27–29,31–33,38,43,44^; notably, this includes surveys of genetics HCPs.^31,33^ The most commonly cited reason for wanting SF was to have the opportunity to act on findings, although the definition of “actionability” varied. It included the availability of treatment^13,24,33,36,42,45,46,47,48^ and prevention,^24,25,26,42^ but others endorsed or raised issues such as the ability to plan or alter lifestyle^24,26,27,32,35,41,42,45,49^ or influence reproductive decision making.^25,29,30,32,33,37,45,48,50,51^ More than half of stakeholders, to whom the question was asked, desired to receive or return “all” secondary findings, regardless of actionability (52–100%).^25,26,27,28,33,38,39,43^ Some cited knowledge as empowering,^25,29,32,41^ and many felt a sense of entitlement to this information (detailed further under “Rights and Responsibility” below). Of note, while some studies reported that “all” study participants were interested in receiving some form of SF,^23,25,29^ many reported a small proportion of recipients who wanted only primary findings.^27,28,31,33,38,41,44^ Providers did support the return of some SF; however, comparative studies found slightly less support for returning SF among genetics HCPs compared to nongenetics HCPs,^33^ and among providers with more clinical training.^42,52^ These are professionals who interact directly with patients and might have disclosed, or be in a position to disclose, SF. Support for return of SF with less certainty (including variants of unknown significance) was lower than unambiguously pathogenic variants, but it was still present.^13,24,29,41,48,50^

*Impacts and implications.* Inevitably, as a result of the emergent use of WES/WGS, discussions of impacts of SF disclosure from WES/WGS were largely hypothetical. The most commonly cited reason against disclosure was the potential to cause anxiety or psychological harm.^24,29,34,35,36,41,43,45,46,49,50,53^ This theme was widely raised in qualitative studies (**Table 1**): semi-structured interview and focus group designs elicited interactive and qualified opinions, thus providing greater insights into how SF might be construed.^30,32,39,40,41,42,46,49,50^ In some of these studies, participants acknowledged becoming less inclined to desire all SF during the course of the interview or focus group.^23,39,46^ Although recognized by both WES/WGS providers^34,43,49,50,51,53,54,55^ and recipients,^26,39,41,46,49^ potential psychological harms of SF were more frequently of concern to providers. Recipients discussed the burden of knowing, particularly about disease risk that may not affect them for some years, the potential for SF to adversely change the way they lived their lives, and incompatibility with religious beliefs.^30,35,40,41,46^ One study found that nearly three-quarters of participants factored potential distress arising from SF into their decision^26^; however, for most, potential anxiety did not override their desire to receive SF.^24,34^

Stakeholders also raised concerns about overwhelming WES/WGS recipients with too much information,^54^ discrimination in insurance and employment,^24,26,30,32,36,41,56^ privacy,^23,27,41,44,51,54^ and stigmatization.^41,48,50^ WES/WGS providers, specifically, raised the following additional concerns: justice issues related to limited resources,^45,48,49,57^ complex logistics^57,58^ strains on time and funding,^45,48,57,58,59^ lack of participant understanding,^48,60^ qualification of HCPs to manage SF,^48^ and the fact that knowledge around WES/WGS is currently limited and still developing.^47,48,57^ Providers were concerned that disclosure of actionable SF would warrant treatment or surveillance that may be harmful in itself or expensive.^45^ It may not always be clear how follow-up might be funded, particularly in insurance-based health-care systems.^48,57^

*Literacy.* Providers were often concerned that WES/WGS recipients do not always appreciate the implications of SF and may not be adequately informed to make decisions.^45,47,48,60^ WES/WGS providers who have consented patients to WES/WGS considered that participants' lack of experience with the conditions related to SF complicates decision making.^61^ Providers felt that recipients' apparent enthusiasm for a wide range of SF—often wider than most programs currently offer—may result from incomplete understanding of the implications of SF.^47^ Studies did report highly variable knowledge of WES/WGS among WES/WGS recipients, both potential and actual,^26,27,33,35,43,44,56^ and providers' own understanding of SF was highly variable and sometimes insecure,^13,50,55,58,62,63^ with implications for informed consent provision.

*Pretest processes.* Stakeholder groups, across many studies, felt strongly that disclosure of SF should be guided by decisions made during consent.^24,34,45,58,60,64,65^ To optimize informed decision making, stakeholders in both groups felt that pretest discussions should supplement written information.^24,49,64^ Most providers felt that these discussions should include SF, whereas others variably included which SF would be reported, false-positive/negative results, changing the interpretation of variants, family, and the potential for anxiety.^54,61,62^ Discussions are very context-dependent and should be flexible and tailored to the recipient.^45,47,54,56,61^ Very recent studies of HCPs' experiences with WES/WGS counseling highlighted often-unrealistic recipient expectations and the need to counteract this.^43,56,61^ The length of time required to achieve informed consent was seen as burdensome for providers and recipients,^56,61^ but some practitioners described increasing confidence in efficiently and effectively obtaining informed consent with experience.^45,61^ Actual WES/WGS recipients and HCPs who have consented them acknowledged recipient difficulty in thinking about SF during consent discussions when they are preoccupied by their primary health condition.^23,61^ Despite this, one qualitative study involving actual WES/WGS recipients found little support for a second consent process after the report of primary findings.^23^

*Posttest processes.* Members of all stakeholder groups stressed the importance of genetic counseling at disclosure of results. Face-to-face meetings with a professional who has knowledge about genetics and the implications of SF were seen as essential by WES/WGS recipients^27,32,49^ and providers.^47,49,51,58^ WES/WGS providers felt that disclosure discussions should take disease burden into account^34^ and should be tailored to the participant, both in content and timing.^45,54,62^ A quantitative study exploring counseling experience for WES/WGS found that although both varied greatly in length, posttest discussions often lasted longer than pretest discussions.^62^ WES/WGS recipients also believed that a plan of action for clinical follow-up should be discussed at the point of disclosure,^24,30^ and some envisaged a need for ongoing support.^30^ Some HCPs felt they would not be able to provide psychosocial support themselves and would also require support from specialists specific to the SF disclosed.^43,63^

*Family.* Relatives are considered important in the management of SF and a crucial component of informed consent and SF disclosure.^25,34,35,42,47,57^ Some studies of providers highlighted difficulties with family-based recruitment and consenting,^54,56^ and several studies echoed issues well described in genetics regarding confidentiality and the right not to know.^32,42,47,49,64,65^ Some recipients cited family as motivation to receive SF and, particularly for healthy individuals, for initial participation in WES/WGS.^25,34,40,41,48^ Stakeholders across all groups are, in principle, willing to share their personal WES/WGS results.^26,27,30,32,34,35,40,44,45,46,54,65^ However, despite wanting SF themselves, some recipients might filter SF information as follows: sharing only actionable findings,^26,27,49,65^ making decisions about impact on individual relatives,^27^ waiting until asked about results,^45^ or being selective about which relatives to tell.^26^ One study raised the potential for parents to experience guilt about the implied risk of an SF to descendants.^46^

*Rights and responsibility.* The concepts of rights and responsibilities are strongly represented across the included studies. Potential WES/WGS recipients stressed the importance of autonomy—the right to choose which SF (if any) they should receive.^24,26,27,30,32,39,41,46,49^ These stakeholders also expressed a strong sense of ownership and desire for control over their genetic information.^24,26,27,30,36,41,46,48,49^ This sense of proprietorship even extended to family genetic data, with some stakeholders expressing a right to be informed about genetic findings present in relatives.^26,42,47^ Perceived rights to “their” information appeared to be an important driver for potential recipients' desire for a wide range of SF: there were strong objections to paternalism, or WES/WGS providers withholding or interpreting information on recipients' behalf, in an effort to decide what is in their best interest.^13,24,49^

WGS providers agreed that participant autonomy was important, and most believed they would respect consent decisions with respect to the SF offered.^13,27,30,42,45,47,49,50,51,54,55,58,59,60,64^ However, providers also perceive a strong sense of responsibility to recipients and believe that fulfilling this requires careful consideration about the utility of SF, often supporting a multidisciplinary approach to reach optimally informed disclosure decisions.^42,47,58,60^ Providers in one study raised concerns about determining the significance of individual variants, listing various sources of evidence (e.g., allele frequency, segregation data, and previous reports) contributing to judgments, but that, ultimately, pathogenicity is often very difficult to assess.^57,60^ Providers felt this uncertainty would be challenging for patients to understand.^48^ Responsibility was also discussed with respect to SF management in general, with most groups agreeing that responsibility must be shared between all parties^24,26,49,50,57,58,60,63,65^: HCPs, WES/WGS recipients,^24,41,48,57,60^ parents,^26,32,36,46,49^ and laboratory professionals.^50,58^

In some studies, providers' views were derived from experience with other genetic testing methods (such as prenatal or microarray-based testing) and note patients' mixed and complex reactions, unanticipated before testing, to receiving SF.^47,50^ When WES/WGS recipients choose not to receive SF, some providers foresee a possible conflict between their professional responsibility in the duty to warn and respecting participant autonomy.^50,58,60^ Some providers considered over-riding a decision not to receive SF if a clinically significant SF were found.^13,42,47,48,50,54^

*Time.* The idea of “duration of responsibility” recurred in various studies and stakeholder groups. Most WES/WGS providers supported gaining consent for re-interrogation of data and re-contact as new interpretations become available.^13,32,54,60^ Potential recipients also felt that they should be re-contacted as interpretation changes or information becomes relevant, such as approaching the age at which a screening program might begin.^29,32,39,46^ However, there was no consensus as to who should initiate re-contact; some felt WES/WGS recipients should share this responsibility with providers.^49,60^ WES/WGS providers did consider that the duration of responsibility might differ depending on whether the program had a research or clinical focus: researchers felt that their responsibility would have to be restricted to the duration of funding^65^ and clearly communicated during the consent process.^55^ Some providers had concerns that the loss of connection after the completion of a research study would complicate re-contact in a research setting.^54^

*Policies and practices.* Stakeholders in some studies appreciated the logistic complexity of SF management, and that it is further complicated by incomplete understanding of significance for current and/or future health.^23,47^ Some raised concerns about the quality and accuracy—in terms of both analytical and clinical validity—of current sequencing technologies and analysis^39,48,55,57^ and worried that there are currently insufficient resources to accurately analyze the data and return it in a meaningful way.^49^ Many HCPs and genomics researchers were unaware of definitive local procedures, policies, or expectations regarding WES/WGS and SF.^47,50,53,57,63,65^ WES/WGS providers discussed various options for SF management. There was support for the ACMG “minimal gene list,”^51,58,59^ “binning” or categorizing of variants,^48,60^ an advisory board or multidisciplinary team,^58,60^ and for minimizing the generation of SF at the present time.^47,49,64,65^ However, there was no consensus on the best approach, and other study participants voiced arguments against each of these options.^47,48,58,59,60^ There was agreement that guidelines should be flexible, allowing for varying levels of expertise in a highly dynamic field.^42,46,47,50^

### Synthesis

**Figure 2:**
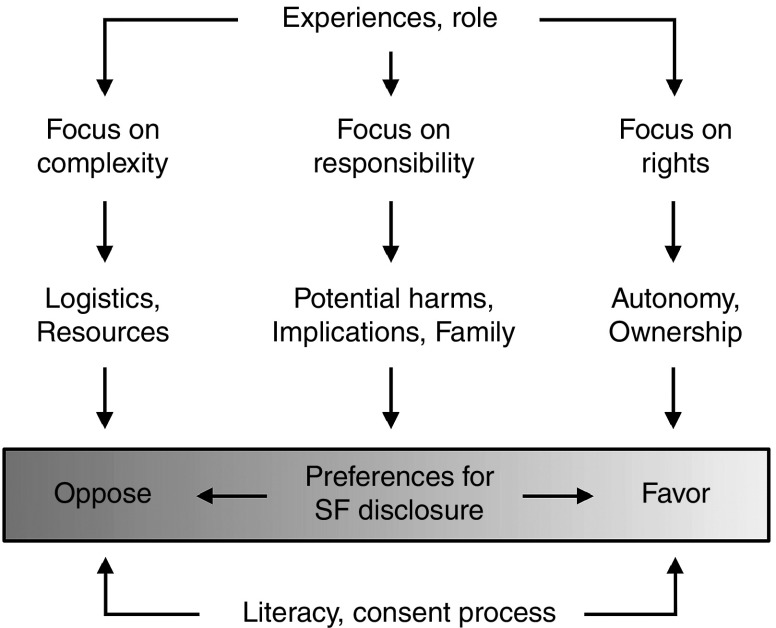
**Theoretical framework illustrating factors influencing stakeholder preferences regarding disclosure of secondary findings in whole-exome and whole-genome sequencing.**

There is a spectrum of views about SF management, ranging from generating and disclosing a wide range of SF to intentionally minimizing their occurrence. **Figure 2** offers a theoretical framework that illustrates the balancing of factors that influence stakeholder preferences around SF disclosure in WES/WGS.

Potential WES/WGS recipients' views frequently fall to the less conservative end of the spectrum, favoring disclosure of a wide range of SF often including nonmedical findings as well as those that have health implications but are not medically actionable. This prompts questions about recipients' understanding of genomic complexity and difficulties distinguishing “normal” variation from that which may be clinically significant. Preference for SF among recipients appears to be strongly associated with a sense of rights, such as *ownership* (the right to their personal information) and *autonomy* (the right to choose what information they receive). Potential recipients feel that they are entitled to access their genetic results if they choose to receive them and perceive genomics/health-care professionals' deliberations and restriction of SF as paternalistic. This review highlights evidence from qualitative studies indicating that recipients' stated preference for SF may be an initial response that might be subject to change with discussion and reflection on possible implications.

Providers' views are also informed by their professional responsibility to WES/WGS recipients. This sense of responsibility manifests in the views about SF; concerned about logistics, including the analysis process and interpretation of variants, and uncertainty about how to handle uncertain results, providers expressed concerns about causing psychological harm by returning SF. Some studies involving HCPs contain quotes suggesting that they have personal anxieties about disclosing findings of potentially high impact^57,60^ or are concerned about the range of possible SF,^63^ raising a possible need for further training and support for HCPs actively disclosing SF.

Studies involving parents highlighted distinct and instructive views.^26,30,32,35,36^ As with other potential recipients, parents wish to receive a wide range of SF but demonstrate intentions to filter the information they tell their children, even when they are no longer minors. This suggests that parents balance a perceived right to their (child's) genomic information with the responsibility to protect their children from any potential harm—analogous to the balancing of benefits and harms exhibited by HCPs. Also of interest, patients with cancer diagnoses were less likely to want a wide range of SF, preferring to receive only clinically actionable findings.^27,29,35^ This may stem from a contextualized understanding of the implications of genetic results for personal health care, based on their experience with genetic testing and illness.

Some studies postulate that greater or lesser knowledge of WES/WGS might account for the differing views. However, many of those who provided insightful responses often reported low “knowledge” of sequencing technology. These individuals, however, frequently drew on their experience with genetic disease.^27,29,41^ For instance, in a qualitative study of patients with Lynch syndrome, only one participant had ever heard of WES/WGS, yet many demonstrated a very nuanced appreciation of findings from WES/WGS.^27^ Across the studies, for participants who had experience with genetic illness and prior genetic counseling and testing, this informed their understanding of possible results and they expressed tolerance for uncertainty. Members of these groups had a broad understanding of (medical) genetics but not necessarily of sequencing technologies, and they were more likely to make distinctions between the kinds of SF they wished to receive. Together with genetics HCPs, WES/WGS recipients with experience with genetic testing for rare diseases or cancer also appreciated that the relevance of SF is dependent on time and circumstances.^27,29,41^

## Discussion

We have presented a systematic review and synthesis of the range of perspectives on secondary findings in WES/WGS. The review found that there is agreement across stakeholders regarding the importance of patient autonomy in the management of SF. Which results should be generated and made available, however, is less clear. Participants generally support the return of “actionable” findings; however, definitions of “actionability” varied. Stakeholders generally agreed that SF for life-threatening diseases for which treatment or surveillance is available and actionable should be offered. However, study participants variously included management, lifestyle modifications, and personal utility as aspects of SF “actionability.” Of note, a recent systematic review found no evidence that genetic risk estimates motivate behavioral changes.^66^ Nonetheless, this variability highlights the context dependency of “actionability” in SF and poses a challenge to reaching a consensus on SF disclosure and management.

Despite differing views on what to disclose, stakeholders agreed on the concept of shared responsibility regarding SF disclosure. Because there is widespread agreement that SF decisions at the time of WES/WGS consent should determine which results are disclosed, it is incumbent on providers to ensure that WES/WGS recipients are prepared for results, including implications and limitations. Education and greater understanding of the potential and limitations of WES/WGS and SF would support informed decision making and narrow the gap between recipients' expectations and the current situation in which providers consider which SF are useful to generate based on existing knowledge and resources. This remains a subject of debate^67^ and is likely to remain so for the foreseeable future as data accumulate on variant pathogenicity.

Exactly what is required to reach a sufficient level of understanding about SF is not yet clear. It appears that genetic literacy does inform stakeholder decisions and that greater understanding often results in greater circumspection about SF. This “literacy,” however, appears to be less so technical knowledge of WES/WGS and more so qualitative understanding of the implications and limitations. In two recent studies that explore HCP experiences consenting WES/WGS recipients, providers acknowledged a shift in the content of their pretest discussions with experience, that is, moving away from discussing fine details of WES/WGS to focusing on the potential results and implications.^42,61^ It will be important for future studies to find ways of measuring what is important to understand to make informed decisions about SF.

It is worth noting the methodologies used by the primary studies. The current perception in WES/WGS practice that recipients want a wide range of SF is informed by survey studies; presented with a tick box, recipients elect in favor of receiving all SF they are offered (and might wish for more than offered).^25,33^ However, qualitative studies highlight how recipients might alter these initial thoughts later or when they have the chance to reflect on and discuss possible implications. If consent mirrors survey studies in the sense that unidirectional imparting of information is followed by a “tick box” answer, then individuals can be expected to elect in favor of receiving SF. The inclusion of qualitative and quantitative studies in this review allows informative comparison and suggests that for recipients to reach a holistic understanding of the implications of WES/WGS and SF, a bidirectional interaction akin to qualitative methodologies is more likely to facilitate informed decision making. Included studies suggest that participant reflection and discussion can result in greater appreciation of the potential implications and consequences of SF; this is consistent with the fact that fewer people participate in predictive genetic testing, even for conditions for which intervention is available, than express a hypothetical intent.^68^ It is likely that a one-time decision about SF may not represent WES/WGS recipients' changing views and circumstances, and more dynamic forms of consent should be considered.

Stakeholder preference is, by no means, the only factor requiring consideration in policy development regarding SF; further empirical research collecting data on returned SF is essential to inform stakeholder views on clinical utility and impacts in the broadest sense on WES/WGS recipients, health-care systems, and society. Impacts on resources for informed consent and genetic counseling in WES/WGS, for analysis and validation, and for clinical screening and management after disclosure need to be assessed as part of setting overall health-care priorities.

WGS/WES initiatives are taking diverse approaches toward the issue of secondary findings in WES/WGS. This diversity, per se, represents the views of providers and the project-level discussions that have contributed to policy. It is likely that the ACMG list has influenced initiatives that have begun since its publication, with large-scale programs such as the UK 100,000 Genomes Project offering opportunistic screening of a (more limited) gene list in addition to recessive and X-linked carrier status for couples and female participants, respectively. Some protocols offer an even wider range of SF,^69^ whereas others avoid SF generation.^70^ There is a possibility that WES/WGS recipients and the public will view such policy-level decisions as paternalistic, and it will be important for programs to be flexible and responsive to research findings.

## Study limitations

No studies report on experiences with disclosure or receipt of secondary genomic findings. Both WES/WGS recipients and providers are diverse in terms of experiences with genetic illness and testing or professional experience with patients and/or WES/WGS. Although studies of participants with direct WES/WGS experience, particularly recipients, add qualitatively to this dataset, we found no evidence from our analysis that WES/WGS experience alters the thematic areas presented here.

Discussions of return of results often include SF without referencing them explicitly. We have used broad search terms to capture variable nomenclature used by primary studies and a thorough screening process; however, because we excluded non-English studies and articles without full text, and as a result of the variation in terminology around SF, it is possible that we missed some relevant studies.

The included studies predominantly present the perspectives of whites in the United States. Two studies that included stakeholders from other groups^40,47^ presented additional data and indicated that findings to date might not be comprehensive and representative of diverse groups.

## Conclusions and implications for practice

In this systematic review, we have shown that the views of both WES/WGS providers and recipients with respect to returning SF exist on a spectrum. We suggest that a nuanced understanding of the implications of WES/WGS and potential findings—rather than technical knowledge—might better inform the decision making in the management of SF. Views and preferences of providers and recipients are not fixed; they are continually informed by experience, discussion, time, and circumstances. Pretest discussions with trained practitioners are therefore essential to inform participant decisions. We recommend the following:

1. Further research into:
  i. The impacts of SF when returned at the individual and family levels and on health-care systems.
  ii. Stakeholders' appreciation for the implications of WES/WGS and SF, what knowledge is required to make informed decisions about SF, how to assess this, and how they relate to decisions to receive or disclose SF.
  iii. Wider perspectives on SF and WES/WGS, particularly among non-Americans and cultural and ethnic minorities, and individuals undergoing WES/WGS, including parents.
2. Evidence-based training and education for HCPs providing informed consent.
3. Training and support for HCPs returning high-impact SF from WES/WGS.
4. Clarifying expectations and guidelines for translational genomics research.
5. Enhancing communication and apportioning of responsibility between those involved in WES/WGS: HCPs, laboratory professionals, and WES/WGS recipients.

## Disclosure

The authors declare no conflict of interest.

## Figures and Tables

**Table 1:**
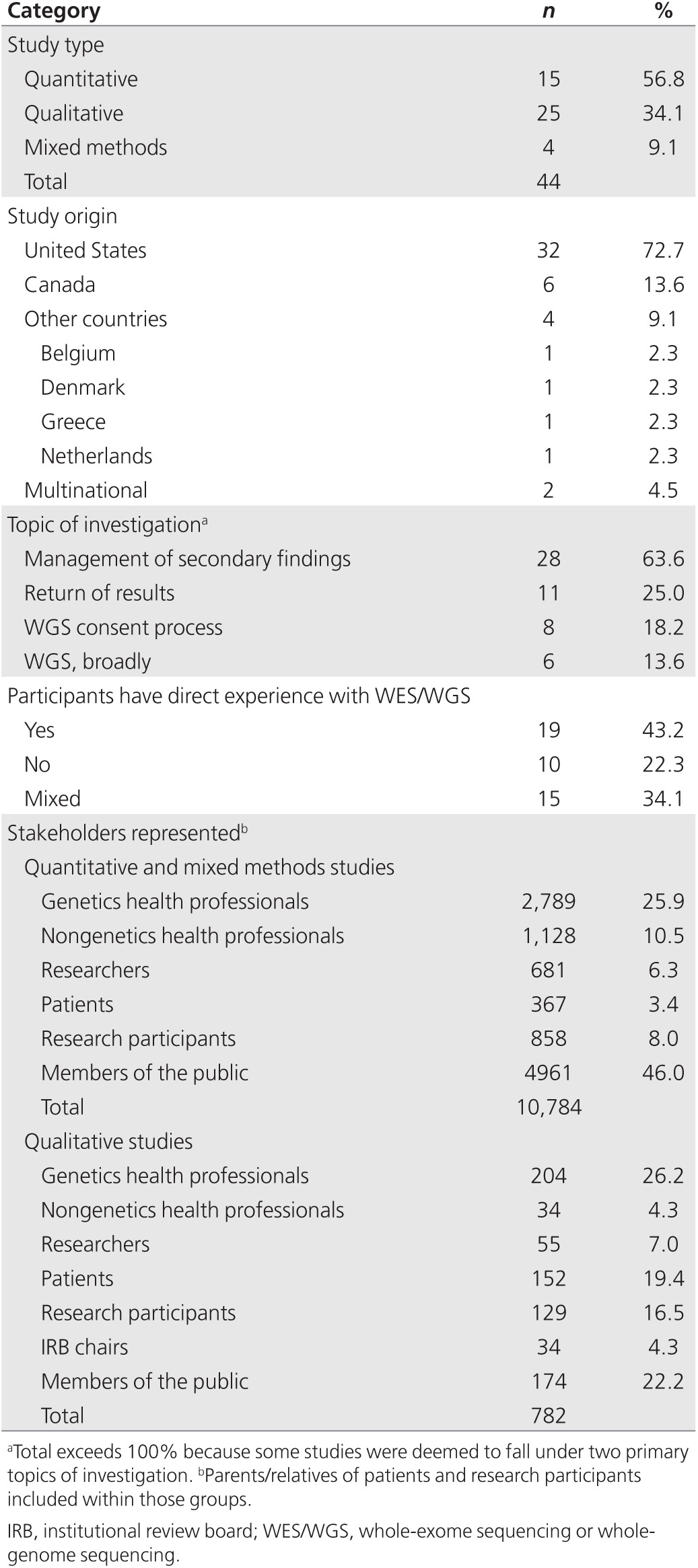
Demographics of included studies

**Table 2:**
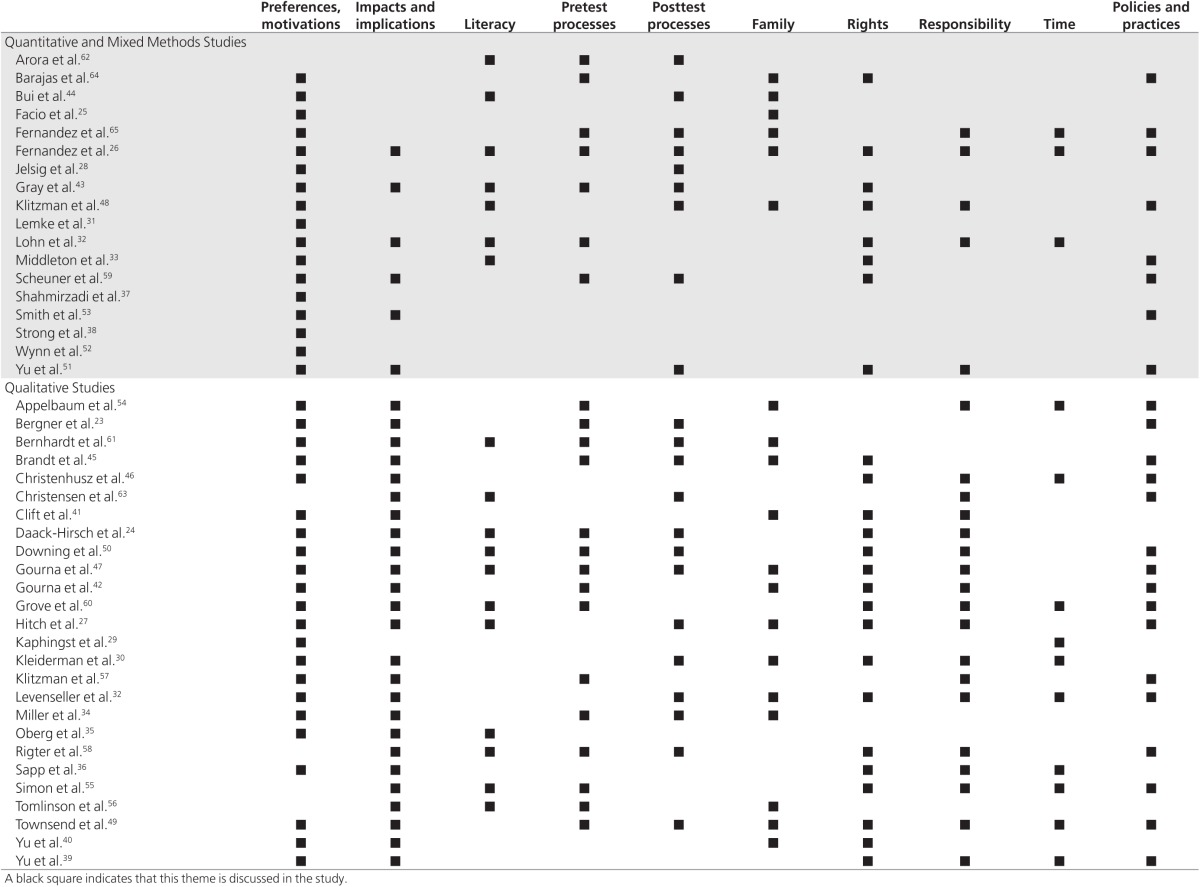
Themes discussed in each study
