# Supplementary material for: Stakeholder views on secondary findings in whole-genome and whole-exome sequencing: a systematic review of quantitative and qualitative studies
Source: Genet Med. 2016 Sep 1;19(3):283–93. doi: 10.1038/gim.2016.109 (PMC5447864; doi:10.1038/gim.2016.109)
Supplement: Supplementary Information [file gim2016109x1.zip › Mackley_SystematicReview_Table S7.docx]

Table 1. Characteristics of included studies

| Author/year | Country | Principal  views or experiences investigated | Study Population | Participant direct experience with WGS | Domain | Design | Primary Topic |
| --- | --- | --- | --- | --- | --- | --- | --- |
| Appelbaum et al.^55^ | USA | Views of informed consent process for SF | 28 genomics researchers*  20 research participants | Mixed | Research | Qualitative,  online survey and semi-structured interviews | WGS consent  Management of SF |
| Arora et al.^63^ | USA | Experiences providing WGS | 49 genetics healthcare professionals | Yes | Clinical | Quantitative,  online survey | WGS  WGS consent |
| Barajas et al.^65^ | USA | Attitudes towards opportunistic screening in pediatric genome sequencing | 179 physicians (bioethicists and pediatricians) | Mixed | Clinical | Quantitative,  email postal survey | Management of SF |
| Bergner et al.^23^ | USA | Experience of the consent process in WGS | 15 research participants | Yes | Research | Qualitative,  Semi-structured interviews | WGS consent process |
| Bernhardt et al.^62^ | USA | Experiences obtaining informed consent for WGS | 21 genetic counselors*  8 research coordinators* | Yes | Both | Qualitative,  semi-structured interviews | WGS consent process |
| Brandt et al.^46^ | USA | Perceived importance of recommended criteria when applied to return of SF | 50 genetics healthcare professionals (13 medical geneticists, 17 genetic counselors/RNs, 20 lab professionals)* 19 genomic researchers 34 IRB chairs* | Mixed | Clinical | Qualitative,  Structured interviews | Management of SF |
| Bui et al.^45^ | USA | Attitudes toward participating in and receiving results from genome sequencing | 58 patients with psychiatric disorders, or unaffected family members | No | Research | Quantitative,  telephone survey | Return of results |
| Christenhusz et al.^47^ | Belgium | Views on SF and their communication | 25 members of public (19 parents, 6 grandparents)  25 genetics professionals (5 laboratory staff, 7 bioinformaticians, 13 genetics students) | No | Clinical | Qualitative,  Focus groups | Management of SF |
| Christensen et al.^64^ | USA | Preparedness around providing WGS | 11 primary care providers  9 cardiologists | No | Both | Qualitative,  Semi-structured interviews | WGS |
| Clift et al.^42^ | USE | Preferences towards return of SF in WGS | 38 patients and parents of patients | Yes | Clinical | Qualitative,  Semi-structured interviews | Management of SF |
| Daack-Hirsch et al.^24^ | USA | Views on SF from genome sequencing in clinical and research situations | 63 members of public | No | Both | Qualitative,  Focus groups and interviews | Management of SF |
| Downing et al.^51^ | USA | Preferences regarding management of SF | 50 genetics healthcare professionals (13 medical geneticists, 17 genetic counselors/RNs, 20 lab professionals)* | Yes | Clinical | Qualitative,  Structured telephone interviews | Management of SF |
| Facio et al.^25^ | USA | Attitudes towards learning results from genome sequencing | 311 research participants | Yes | Research | Mixed methods,  in-person survey | Return of results |
| Fernandez et al.^66^ | Canada | Attitudes towards disclosure of clinically significant research findings in context of regulatory guidance | 74 genomics researchers | Yes | Research | Quantitative,  postal questionnaire | Return of results  Management of SF |
| Fernandez et al.^26^ | Canada | Attitudes towards return of targeted and incidental genomic research results in setting of pediatric cancer, inherited childhood diseases. | 362 parents of research participants | Yes | Research | Quantitative,  postal questionnaire | Return of results Management of SF |
| Gourna et al.^48^ | Greece | Attitudes towards clinical sequencing and return of SF | 10 genetics healthcare professionals (3 clinicians, 2 bioethicists, 5 geneticists)* | Yes | Clinical | Qualitative,  In-depth interviews | Management of SF |
| Gourna et al.^43^ | Multi-national | Attitudes towards return of SF in a clinical setting | 30 genetics healthcare professionals (10 clinicians, 6 genetic counselors, 8 laboratory professionals, 6 legal/ethical specialists)* | Yes | Clinical | Qualitative,  In-depth interviews | Management of SF |
| Gray et al.^44^ | USA | Views on WGS and SF | 167 patients  27 oncologists | Yes | Clinical | Qualitative and quantitative, semi-structured interviews and surveys | Management of SF  WGS |
| Grove et al.^61^ | USA | Experiences surrounding SF in clinical sequencing, views on current policies/guidelines | 35 members of ASHG and/or NSGC (genetics healthcare professionals) | Mixed | Clinical | Qualitative,  Focus groups | Management of SF |
| Hitch et al.^27^ | USA | Views on WGS, return of genomic results | 19 patients who had undergone WES for Lynch Syndrome | Yes | Clinical | Qualitative,  Semi-structured interviews | Return of results |
| Jelsig et al.^28^ | Denmark | Attitudes towards disclosure of SF, and types of SF | 127 research participants | Yes | Research | Quantitative,  unclear design | Management of SF |
| Kaphingst et al.^29^ | USA | Views on return of SF among women diagnosed with breast cancer | 60 women diagnosed with breast cancer under 40 years old | No | Clinical | Qualitative,  Semi-structured interviews | Management of SF |
| Kleiderman et al.^30^ | Canada | Parental perceptions and experiences regarding the return of SF in pediatric research | 15 parents of children with rare disease | No | Research | Qualitative,  Focus groups and interviews | Management of SF |
| Klitzman et al.^49^ | USA | Practices attitudes towards return of secondary findings | 241 genetics researchers*  28 genomics researchers* | Mixed | Research | Multiple methods,  internet survey,  Semi-structured interviews | Management of SF |
| Klitzman et al.^58^ | USA | Experiences and views concerning return of SF | 28 genomics researchers* | Mixed | Research | Qualitative,  Semi-structured interviews | Management of SF |
| Lemke et al.^31^ | USA | Attitudes towards genome sequencing and secondary findings | 279 genetics healthcare professionals | Mixed | Clinical | Quantitative,  internet survey | Management of SF |
| Levenseller et al.^32^ | USA | Views of for the future implementation of WES | 22 genetics healthcare professionals (clinicians, bioethicists, lab directors, and genetic counselors) 20 parents 7 adolescents | Mixed | Clinical | Qualitative,  Focus groups | WGS |
| Lohn et al.^32^ | Canada | Views on the management of SF in clinical context | 210 genetics healthcare professionals | Mixed | Clinical | Mixed methods,  online questionnaire | Management of SF |
| Middleton et al.^33^ | Inter-national | Attitudes towards returning SF from genome research | 4961 members public,  533 genetics healthcare professionals,  843 non-genetic healthcare professionals,  607 genomics researchers | Mixed | Research | Quantitative,  internet survey | Management of SF |
| Miller et al.^34^ | Canada | Expectations and experiences of patients and providers who participate in WGS | 29 patients 14 oncologists | Yes | Research | Qualitative,  Semi-structured interviews | WGS Return of results |
| Oberg et al.^35^ | USA | Challenges to informed consent in pediatric oncology research | 25 parents (15 parents of children with cancer, 10 parents of children without cancer) | No | Research | Qualitative,  Focus groups and semi-structured interviews | WGS consent |
| Rigter et al.^59^ | Nether-lands | First experiences with WGS consent process | 11 genetics healthcare professionals (3 clinical geneticists, 3 molecular geneticists, 2 ethicists, 1 legal expert, 1 quality manager, 2 reps from Dutch Genetic Alliance) | Yes | Clinical | Qualitative,  Semi-structured interviews | WGS consent process |
| Sapp et al.^36^ | USA | Preferences on receiving four different types of results from WES | 25 parents of children with rare genetic disease | Yes | Research | Qualitative,  Semi-structured interviews | Return of results |
| Scheuner et al.^60^ | USA | Attitudes towards secondary findings in clinical genome sequencing | 492 members of the ACMG (genetics professionals) | Mixed | Clinical | Quantitative,  internet survey | Management of SF |
| Shahmirzadi et al.^37^ | USA | Preferences for secondary findings from consent forms | 200 patients who underwent whole exome sequencing | Yes | Clinical | Quantitative,  review of consent forms | Management of SF WGS consent |
| Simon et al.^56^ | USA | How genomic SF should be addressed in informed consent processes | 34 IRB Chairs* | Mixed | Research | Qualitative,  Semi-structured interviews | WGS consent process Management of SF |
| Smith et al.^54^ | USA | How ACMG recommendations have influenced practice | 46 genetic counselors | Yes | Clinical | Quantitative,  internet survey | Management of SF |
| Strong et al.^38^ | USA | Views on return of SF from WGS | 258 primary care providers | No | Clinical | Quantitative,  internet survey | Management of SF |
| Tomlinson et al.^57^ | USA | Challenging experiences in obtaining informed consent for WGS | 21 genetic counselors*  8 research coordinators* | Yes | Both | Qualitative,  semi-structured interviews | WGS consent process |
| Townsend et al.^50^ | Canada | Attitudes towards disclosure of SF in clinical settings | 10 genetics healthcare professionals (3 physician geneticists, 3 genetic counselors, 4 laboratory geneticists), 8 parents  10 members of the public | Mixed | Clinical | Qualitative,  Focus groups | Management of SF |
| Wynn et al.^53^ | USA | How views towards SF in genomics are influenced by professional background and experience | 241 genetics healthcare professionals* | Mixed | Research | Quantitative,  internet survey | Management of SF |
| Yu et al.^40^ | USA | Attitudes towards participation in WGS and return of results | 41 African American members of the public | No | Research | Qualitative,  Focus groups | Return of results |
| Yu et al.^39^ | USA | Attitudes towards participation in WGS and return of results | 35 Non-African American members of the public | No | Research | Qualitative,  Focus groups | Return of results |
| Yu et al.^52^ | USA | Attitudes toward return of SF from WGS | 760 genetics healthcare professionals | Mixed | Clinical | Quantitative,  internet survey | Management of SF |

(-), unclear or unable to ascertain

(*), study population reported in multiple articles
