# Supplementary material for: Stakeholder views on secondary findings in whole-genome and whole-exome sequencing: a systematic review of quantitative and qualitative studies
Source: Genet Med. 2016 Sep 1;19(3):283–93. doi: 10.1038/gim.2016.109 (PMC5447864; doi:10.1038/gim.2016.109)
Supplement: Supplementary Information [file gim2016109x1.zip › Mackley_SystematicReview_SupplementaryMaterial_S1.pdf]

S1 Systematic search strategy on PubMed

01. incidental finding*
02. secondary finding*
03. additional finding*
04. incidental genetic finding*
05. 1 or 2 or 3 or 4
06. clinical sequenc*
07. genetic sequenc*
08. genomic sequenc*
09. genome sequenc*
10. exome sequenc*
11. 6 or 7 or 8 or 9 or 10
12. 5 and 11

(“incidental finding*” OR “secondary finding*” OR “additional finding*” OR “incidental genetic finding*”) AND (“clinical sequenc*” OR “genetic sequenc*” OR “genomic sequenc*” OR “genome sequenc*” OR “exome sequenc*”)
