# Supplementary material for: Stakeholder views on secondary findings in whole-genome and whole-exome sequencing: a systematic review of quantitative and qualitative studies
Source: Genet Med. 2016 Sep 1;19(3):283–93. doi: 10.1038/gim.2016.109 (PMC5447864; doi:10.1038/gim.2016.109)
Supplement: Supplementary Information [file gim2016109x1.zip › Mackley_SystematicReview_SupplementaryMaterial_S2S3.pdf]

S2 Quality Checklist for Quantitative Studies

I. Are the aims of the research clear?*
II. Is the research relevant to the synthesis topic?*
III. Was a questionnaire survey an appropriate research design to answer this question?*

*If "No" to any of I-III, paper is to be omitted from synthesis

Sampling

1. *Sampling frame:* Is the sampling frame clear, and was it sufficiently large and representative?
2. *Understanding and suitability:* Does it appear that all participants understood what was required of them, and did they attribute the same meaning to the terms in the questionnaire? Does the instrument appear to take into account the likely range of abilities of potential participants?

Data Collection

3. *Existing measures:* Did the researchers use an existing questionnaire or survey? If not, did they justify their development of a new one?
4. *Consumer views:* Were the views of consumers sought about the questionnaire?
5. *Validity:* Have the authors claimed that the instrument is valid? Is this claim justified? (i.e. Do the authors make it clear that the instrument measures what it sets out to measure?)
6. *Reliability:* Have the authors claimed that the instrument is reliable? Is this claim justified? (i.e. Do the authors make it clear that the instrument provides stable results over time and between researchers?)
7. *Pilot:* Was a pilot used on a representative sample? Was the instrument modified accordingly?

Instrument

8. *Instrument provided:* Has the questionnaire or survey been provided?
9. *Title:* Is the title of the questionnaire provided? If so, is it appropriate?
10. *Sensitivity:* Do the questions cover all relevant aspects of the problem in a non-threatening and non-directive way? (i.e. Are non-threatening questions placed at the beginning, and sensitive ones at the end?)
11. *Types of questions:* Are open-ended and closed-ended questions used appropriately?
12. *Briefness:* Is it clear that the questionnaire was kept as brief as the study allowed?
13. *Clarity:* Do the questions appear clear? (i.e. not ambiguous or overly complicated).
14. *Instructions:* Is it clear that adequate instructions were provided in the instrument? (i.e. examples answers, instructions on how to return).

Distribution, Administration and Response

15. *Method of distribution:* Is it clear how the questionnaire or survey was distributed?
16. *Method of administration:* Is it clear how the questionnaire or survey was administered?
17. *Response rate:* Is the response rate clear and reasonable?
18. *Non-participation:* Have non-responders been accounted for?

Data Analysis

19. *Description of analysis process:* Is the analysis process clearly described?
20. *Appropriateness of analysis:* Is it clear that quantitative data were subjected to statistical analysis and qualitative data to qualitative analysis (if relevant)?
21. *Accuracy measures:* Is it clear that measures were in place to maintain the accuracy of the data?
22. *Free from data dredging:* Does the study appear to be free from 'data dredging'? (i.e. No evidence that statistical analyses that were not 'hypothesis driven').

Reporting

23. *Clarity of findings:* Are the findings explicit? Have all relevant results been reported (significant and non-significant)?
24. *Data and findings consistent:* Were the findings consistent with the data provided?
25. *Appropriateness of reporting:* Are quantitative results presented as definitive (significant, non-significant)? If relevant, have qualitative results been presented with representative quotations?
26. *Relation to current practice and literature:* Have the findings been discussed in relation to current practice or research-based literature?

Checklist adapted from Boynton & Greenhalgh (2004) and Greenhalgh et al. (2005)

S3 Quality Checklist for Qualitative Studies

I. Are the aims of the research clear?*
II. Is the research relevant to the synthesis topic?*
III. Is this qualitative research (i.e. does this article report on findings from qualitative research, and did that work involve qualitative methods of data collection and analysis)? Is a qualitative method appropriate?*

*If "No" to any of I-III, paper is to be omitted from synthesis

Personal Characteristics

1. *Interviewer:* Has the interviewer/facilitator been identified?
2. *Credentials:* Have the researchers' credentials been provided?
3. *Occupation:* Have the researchers' occupations been provided?
4. *Gender:* Have the researchers' genders been provided?
5. *Experience and training:* Have the researchers stated their experience and training?

Relationship with participants

6. *Relationship established:* Is it clear whether or not a relationship was established prior to study commencement?
7. *Participant knowledge of the interviewer:* Did the participant know something about the researcher (i.e. goals, reason for research, etc.)?
8. *Interviewer characteristics:* Have characteristics of the interviewer been reported?

Theoretical framework

9. *Methodological orientation and theory:* Is it clear which methodological orientation underpinned the study (i.e. grounded theory, phenomenology, etc.)?

Participant Selection

10. *Sampling:* Is it clear how participants were selected (i.e. purposive, convenience, consecutive)?
11. *Method of approach:* Is it clear how participants were approached?
12. *Sample size:* Is the sample size given?
13. *Non-participation:* Is it clear how many and/or why some participants chose not to take part?

Setting

14. *Setting of data collection:* Is it clear where the data was collected?
15. *Presence of non-participants:* Was anyone else present besides the participant and researchers?
16. *Description of sample:* Are important characteristics of the sample discussed?

Data Collection

17. *Interview guide:* Has a topic or interview guide been provided?
18. *Repeat interviews:* Have any repeat interviews been conducted?
19. *Audio/visual recording:* Are the data recording methods clear?
20. *Field notes:* Were field notes taken?
21. *Duration:* Is the duration of the interview or focus groups clear?
22. *Data saturation:* Has the researcher discussed data saturation?
23. *Transcripts returned:* Have transcripts been returned to participants for comments/corrections?

Data Analysis

24. *Number of data coders:* Is it clear how many individuals coded the data?
25. *Description of coding tree:* Has a description of the coding tree been provided?
26. *Derivation of themes:* Is it clear how the themes were derived?
27. *Software:* Has software been used to manage the data?
28. *Participant checking:* Were results presented to participants?

Reporting

29. *Quotations presented:* Have respondent quotations been provided?
30. *Data and findings consistent:* Were the findings consistent with the data provided?
31. *Clarity of major themes:* Are the major themes presented clearly?
32. *Clarity of minor themes:* Are diverse cases, or minor themes, discussed?

COREQ Checklist adapted from Tong et al. (2007) using CASP (2013
