# Supplementary material for: Stakeholder views on secondary findings in whole-genome and whole-exome sequencing: a systematic review of quantitative and qualitative studies
Source: Genet Med. 2016 Sep 1;19(3):283–93. doi: 10.1038/gim.2016.109 (PMC5447864; doi:10.1038/gim.2016.109)
Supplement: Supplementary Information [file gim2016109x1.zip › Mackley_SystematicReview_SupplementaryMaterial_S4S5.pdf]

S4 Quality assessment of quantitative and mixed methods papers

Quality Assessment Criteria		Reference 63	65	45	25	66	26	28	44	49	31	13	33	60	37	54	38	53	52
Sampling	1. Sampling frame		■	■	■	■	■	■	■	■	■	■		■	■	■	■	■	■
	2. Understanding	■																	
Data Collection	3. Existing measures								■				■				■		
	4. Consumer views	■							■	■	■	■	■	■				■	■
	5. Validity				■	■	■		■		■		■						
	6. Reliability						■						■						
	7. Pilot						■		■	■	■	■	■	■				■	■
Instrument	8. Instrument provided		■	■		■	■	■	■			■	■				■	■	■
	9. Title		■	■		■	■		■				■				■		
	10. Sensitivity		■	■		■	■	■	■			■	■		n/a				■
	11. Types of questions	■	■	■	■	■	■	■	■	■	■	■	■	■	n/a	■	■	■	■
	12. Briefness		■	■		■	■	■	■		■	■	■		n/a		■	■	■
	13. Clarity		■	■		■	■	■	■		■	■	■	■	n/a		■		■
	14. Instructions		■	■		■	■	■	■			■	■		n/a		■		■
Administration	15. Distribution	■	■	■	■	■	■	■	■	■	■	■	■	■	n/a	■	■	■	■
	16. Administration	■	■	■		■	■		■	■	■	■	■	■	n/a		■	■	■
	17. Response rate		■	■		■	■	■	■	■	■	■		■		■	■	■	■
	18. Non-participation		■	■		■	■	■	■	■	■			■		■	■	■	■
Data analysis	19. Analysis process	■	■	■	■	■	■	■	■	■	■	■	■	■	■	■	■	■	■
	20. Appropriate analysis	■	■	■	■	■	■	■	■	■	■	■	■	■	■	■	■	■	■
	21. Accuracy measures																		
	22. No data dredging	■	■	■	■	■	■	■	■	■	■	■	■	■	■	■	■	■	■
Reporting	23. Clarity of findings	■	■	■	■	■	■	■	■	■	■	■	■	■	■	■	■	■	■
	24. Findings consistent	■	■	■	■	■	■	■	■	■	■	■	■	■	■	■	■	■	■
	25. Appropriate reporting	■	■	■	■	■	■	■	■	■	■	■	■	■	■	■	■	■	■
	26. Practice and literature	■	■	■	■	■	■	■	■	■	■	■	■	■	■	■	■	■	■
	Total	12	19	19	11	20	22	17	23	15	18	19	21	16	8	12	19	17	20

(n/a), not applicable based on the research methodology employed

S5 Quality assessment of qualitative papers, as well as mixed methods papers that employed qualitative methodologies

Quality Assessment Criteria		Reference																													
		55	23	62	46	47	64	42	24	51	25	48	43	44	61	27	29	30	49	58	32	13	34	35	59	36	56	57	50	40	39
Personal characteristics	1. Interviewer					■		■	■		n/a	■	■		■	■		■				n/a		■		■			■	■	
	2. Credentials	■									n/a					■						n/a		■					■	■	
	3. Occupation			■	■			■			n/a							■				n/a									
	4. Gender										n/a											n/a									
	5. Experience										n/a						■					n/a		■							
Relationship	6. Relationship established		■								n/a											n/a				■					
	7. Participant knowledge										n/a											n/a									
	8. Characteristics			■				■			n/a											n/a									
Theory	9. Methodological orientation	■						■							■			■	■	■			■								
Participant selection	10. Sampling	■	■	■	■	■	■		■	■	■	■	■	■	■	■	■	■	■	■		■	■	■	■	■	■	■	■	■	■
	11. Method of approach	■	■	■		■	■	■	■		■	■	■	■	■	■	■		■	■	■	■	■	■	■	■	■	■	■	■	■
	12. Sample size	■	■	■	■	■	■		■	■	■	■	■	■	■	■	■	■	■	■	■	■	■	■	■	■	■	■	■	■	■
	13. Non-participation	■		■			■					■	■	■		■			■	■	■		■		■			■	■	■	
Setting	14. Setting	■	■	■	■			■	■	■	■			■	■	■			■	■		■	■	■	■	■	■	■	■	■	
	15. Others present					■															■								■		
	16. Sample description	■	■	■	■	■	■	■	■	■	■		■	■	■	■	■	■	■	■	■	■	■	■		■	■	■		■	■
Data Collection	17. Interview guide	■			■	■	■		■	■			■	■			■	■		■		■		■		■	■	■	■		
	18. Repeat interviews							■																							
	19. Recording		■	■	■			■	■	■	n/a	■	■	■	■	■	■	■			■	n/a	■	■	■	■	■	■	■	■	■
	20. Field notes										n/a										■	n/a									
	21. Duration	■	■	■	■	■	■	■	■				■	■	■	■	■	■	■	■					■	■	■	■	■		
	22. Data saturation									■	■													■			■		■		
	23. Transcripts returned																								■						
Data Analysis	24. Number of coders	■	■	■	■	■		■	■	■	■		■	■	■	■	■	■	■	■	■	■		■	■	■	■	■	■	■	■
	25. Coding tree					■														■				■		■					
	26. Derivation of themes			■			■	■			■	■			■		■		■	■	■			■					■	■	■
	27. Software	■	■	■	■	■	■	■	■	■	■	■	■	■	■		■	■		■	■			■	■	■	■	■	■	■	■
	28. Participant checking																														
Reporting	29. Quotations	■	■	■	■	■	■	■	■	■	■	■	■	■	■	■	■	■	■	■	■	■	■	■	■	■	■	■	■	■	■
	30. Findings consistent	■	■	■	■	■	■	■	■	■	■	■	■	■	■	■	■	■	■	■	■	■	■	■	■	■	■	■	■	■	■
	31. Major themes	■	■	■	■	■	■	■	■	■	■	■	■	■	■	■	■	■	■	■	■	■	■	■	■	■	■	■	■	■	■
	32. Minor themes	■	■	■	■	■	■	■	■	■	■	■	■	■	■	■	■	■	■	■	■	■	■	■	■	■	■	■	■	■	■
	Total	16	14	17	14	15	13	17	15	13	13	12	15	16	16	15	15	15	14	17	14	11	12	19	14	17	15	15	19	16	12

(n/a), not applicable based on the research methodology employed
