# Supplementary material for: Stakeholder views on secondary findings in whole-genome and whole-exome sequencing: a systematic review of quantitative and qualitative studies
Source: Genet Med. 2016 Sep 1;19(3):283–93. doi: 10.1038/gim.2016.109 (PMC5447864; doi:10.1038/gim.2016.109)
Supplement: Supplementary Information [file gim2016109x1.zip › Mackley_SystematicReview_SupplementaryMaterial_S6.pdf]

S6 Illustrative quotations from the primary studies for each theme

Preferences for secondary findings

- *Affected adult research participant:* "And then as far as discovering other things, I mean, if there's something else going on, I'd like to know now so that if there's treatment or things that I'm doing that I need to stop doing, I'd definitely like to know that."[23]
- *Member of the public:* "It's just all so arbitrary, it's so, so, unbelievably person-dependent and situation-dependent and disease-dependent too, I don't know."[47]
- *Medical genetics specialist:* "If it's not going to impact [the patient], and they didn't ask about it, I probably wouldn't share it"[46]
- *Medical genetics specialist:* "Personal utility has never been the purpose of medicine…I think the idea of actionability, as the idea to change treatment, the medical actionability, is the way to go on with this. This is the way we think in medicine."[43]

Impacts and implications

- *Medical genetics specialist:* "[W]hen these incidental findings come up in a prenatal setting, the anxiety, the turmoil, that I see out of this is tremendous…"[51]
- *Adolescent patient:* "I'd rather be surprised than know it's coming, because that's worse, to me…because if I can't cure it, the anxiety of when it will come would make me sick."[32]
- *Affected adult research participant:* "Like these crazy movies they're based off of that information gets to the wrong people and somehow they do crazy stuff, but I don't see that happening."[23]
- *Researcher:* "The number of potential IFs is essentially infinite. The amount of overhead for identifying and reporting incidental clinical findings would destroy the research enterprise in genetics."[49]

Literacy

- *Researcher:* "Most people don't think hard about the implications. The casual comment "I want everything" is usually not so reflective of what people want once they've really thought and been educated about it."[49]
- *Patient with lynch syndrome:* "Unless you might have something that will impact your health in the future, I don't know if it's good or useful for just anybody to get to see their whole genetic makeup."[27]
- *Genetic counselor:* "I don't think of incidental findings as things like variants of uncertain significance, but that's a different issue altogether, but they sort of get jumbled in my head."[51]

Pre-test processes

- *Genetics healthcare professional:* "I think one of the most important factors is the pre-test consent. Patients have the right to choose if they would like to obtain information about themselves above and beyond information about the pertinent clinical question.

The types of results should be explained to the patient who can then decide what they want or don't want to know."[13]

- *Genetic counselor:* "I mean, you'd have to have a 10-hour counseling session to talk about all of the things you might find."[51]
- *Researcher:* "The problem with the consent process is whether to be as detailed as you want. I'm always very fearful that a subject is just going to turn off and just sign, or just say, 'Forget it,' because we come up with these well-intentioned, but terribly long consent documents."[55]

Post-test processes

- *Researcher:* "You have to tune into the person, what their needs are, and their level of understanding. I tend to err on the side of trying to give them a lesson in what the genetics of this are, and walk them through this as systematically and slowly as I can…It depends a lot on the perception you have of how much the patient is able to understand and wants to understand. We don't handle it exactly the same way every time."[55]
- *Medical genetics specialist:* "I think [it depends on] what the patient is ready to hear…if you've got eight things to discuss you don't have to throw all eight at a patient or a family immediately in the first 10 min of a counseling session and then clean up the mess. I think you can deal with it over time…To prioritize…in a manner that allows the patient or the family to adapt…gain knowledge and then be able to handle something new."[46]
- *Parent of affected child:* "Absolutely verbal. […] It would almost seem uncaring just to receive it on a piece of paper."[30]
- *Parent of child subjected to WGS:* "Sometimes you need these things translated…My concern would be... are all GPs…able to interpret the results of the testing?"[50]

Family

- *Member of the public:* "... the biggest thing... is... physicians having a problem: 'Do I disclose to other family members?' That's where the confidentiality issues arise. If you find something that's definitely inheritable... do you tell everyone? ... What if they don't want to know?"[50]
- *Member of the public:* "I may not want this information but the generations that come after me, Insha'Allah, God willing, could come back and access it."[40]
- *Patient with lynch syndrome:* "I'm someone who can really handle information and not get too freaked out by it, but it's a fine delicate balance of like telling someone if they have something that's completely unrelated to, you know, what I've been going through. I think I'd ask them if they want to know and I would really think about it and probably get the opinion of a genetic counselor."[27]

Rights and Responsibility

- *Pediatric genetics healthcare professional:* "This is the nanny state rearing her goat head again! No one should be forced to undergo testing and receive information that they don't want."[65]

- *Patient eligible for WGS:* “I also heard that someone makes that decision on the basis of ethics and that’s what made me think: Well, aren’t we man enough to decide for ourselves to decide what [unsolicited findings] we think is responsible to hear or not.”[59]
- *Genetic counselor:* "It is absolutely their right to choose…but I think it would be really difficult if we knew something that was medically important, possibly treatable…"[51]
- *Researcher:* "Incidental findings with clinical utility, e.g., BRCA1 and BRCA2, should be disclosed, period, and the participant should be told to expect a call if those show up. They should not be deciding [for themselves and relatives] to ignore it."[49]
- *Genetics healthcare professional:* “I think if you tried to be paternalistic and dictate there were things that we shouldn’t tell them then you may get a lot of push back from patient advocacy groups.”[50]
- *Patient:* “I’m wondering why is it even an issue of the doctor having a say? I mean it’s your genome. It’s your body...”[50]
- *Genetics healthcare professional:* “It is not our role to withhold information but instead to make this information accessible and understandable to our patients. Once reported, it becomes part of those patients’ medical records and therefore information they are entitled to have.”[13]

Time

- *Genetic counselor:* “What I need today from my genome sequencing isn’t what I’m gonna need 5 years from now. And I think about the preventive you know timeline that we look at in the doctor’s office every time you go to see your OB-GYN. When I’m 20-25, I need to have my cholesterol screened once every 5 years. I need to have, so having something like that in the whole genome, what do I need to unlock and what do I need to, do I need to find out about this now? […] What do I want, so some concept like that I think needs to be flushed out.”[61]
- *Patient with breast cancer:* “They still don’t fully understand it, but at some point, hopefully they will. … They would be able to go back and say okay, now we have something we can use with this information.”[29]
- *Genetics healthcare professional:* “You can certainly put the onus on the patient... but I don’t think that you can put the onus on the lab or the physician’s office to retroactively review all of their cases where ‘X’ variant was found because now there is this new information on ‘X’ variant.”[50]

Policies and practices

- *Researcher:* “I would never return, even think of returning something that’s coming out of next-gen[eration] sequencing right now.”[49]
- *IRB Chair:* “The dilemma, of course, is whatever incidental finding has been discovered…it’s never clear how accurate that information is, how correct it is, and whether it is meaningful to the subject.”[56]
- *Clinical molecular geneticist:* “[An advisory board] is of course something that in the long run will be unmanageable. At a certain point in time, we will have to report our

findings within two weeks and if every incidental finding has to pass a committee, that will never work."[59]
